# The Fracture Mechanism of Impact-Resistant Bionic 3D Model Structures Inspired by Composite Structure of Mantis Shrimp Appendage

**DOI:** 10.3390/biomimetics11030162

**Published:** 2026-03-01

**Authors:** Xiao Yang, Miaoyu Meng, Xingyu Meng, Aolong Huang, Chun Shao, Yonghua Wang, Hao Jin

**Affiliations:** 1 School of Mechanical Engineering, Hangzhou Dianzi University, Hangzhou 310018, China; 2 College of Mechanical and Electrical Engineering, Changchun University of Science and Technology, Changchun 130022, China

**Keywords:** fiber composites, impact resistance, bionic structure, multi-directional

## Abstract

To improve the impact resistance of composite materials, this study adopted the structures in the impact region of mantis shrimp appendages as a bionic prototype, designing a composite structure with a rigid outer layer and flexible sinusoidal inner layer. Meanwhile, bionic arrangement was conducted on the fibers in three directions (X-Y, X-Z, and Y-Z planes) within the flexible layer to regulate the crack propagation path during the impact process. Finite Element Method and low-velocity impact tests were carried out to verify the structural effectiveness, analyze the energy absorption mechanism, and investigate the failure modes. Relative to the basic rigid-flexible structure, the brick-and-mortar (Y-Z) and vertical-horizontal alternating fiber (X-Y) models show a 94% and 109% improvement in kinetic energy absorption efficiency, respectively. Additionally, the catastrophic damage in the impact center area caused by crack concentration is significantly reduced. This study confirms that the bionic 3D arrangement of fibers can realize interlayer connection, optimize crack distribution, and enhance energy dissipation, thereby improving the impact resistance of composite materials.

## 1. Introduction

Designing and manufacturing structural materials with properties including high specific stiffness, high energy absorption capacity, corrosion resistance, and fatigue resistance has long been a goal pursued by material scientists [1]. Among these materials, composite materials are widely used in industries such as transportation, aerospace, and national defense, owing to their excellent comprehensive performance. For instance, in the design of automobiles, aircraft and ships [2], composite components at key positions are required to have the ability to resist the impact of various external loads. Notably, structural properties are comparable in importance to material properties; however, in most research or practice, the importance of structural properties often fails to receive adequate attention [3]. With the progressive development of bionics, nature-inspired structural design has emerged as an effective approach. Natural materials are renowned for their high efficiency and have been widely utilized as they can achieve diverse functions—such as structural support and protection, maintaining structural flexibility, and resisting extreme environments—with the minimum amount of material [4]. The appropriate integration of bionic microstructures into traditional composite materials can effectively enhance the strength and toughness of composites, thereby improving their impact resistance [5,6,7]. Over the past few decades, researchers have investigated the brick-and-mortar structure in the nacre of seashells by imitation and adopted it as a model system for toughening bio-composite materials [8]. Nevertheless, the brick-and-mortar structure is vulnerable to attack from a smashing predator known as the mantis shrimp [9]. This observation indicates that numerous traditional bionic structures, typified by the brick-and-mortar structure, still have substantial room for improvement. Consequently, the biomimetic application of more superior biological structures is highly necessary [10,11].

Mantis shrimps are a group of highly aggressive marine crustaceans, whose predatory strategy relies on the high-speed movement of their appendages—they can accelerate from a stationary state to 23 m/s in an extremely short time, using this to shatter the hard exoskeletons of prey such as seashells and crabs [12,13]. The excellent mechanical properties of the mantis shrimp appendage (MSA) are attributed to its ingenious microstructures. From the outside to the inside, the MSA consists of three distinct regions: the impact region, the periodic region, and the striated region. Among these, the outermost layer of the impact region is an impact surface composed of highly mineralized hydroxyapatite (HAP) nanoparticles, which can dissipate impact energy through particle fragmentation and sliding [14,15]. Beneath this layer lies a region made up of mineralized fibers arranged in a sinusoidal pattern, endowing the region with excellent impact resistance. The periodic region features the Bouligand structure, in which the fibers are arranged in a helical pattern and filled with fibrous pore tubules perpendicular to the surface of the MSA [16]. The striated region is composed of directionally aligned chitin fiber bundles; these bundles surround the outer side of the periodic region and can restrict its lateral displacement. With the continuous advancement of characterization techniques and the in-depth study of the MSA, the application of these unique biological structures in the engineering field has become a highly promising strategy. For instance, regarding the Bouligand structure in the periodic region, numerous experiments have confirmed that this structure can extend the crack propagation path, thereby increasing the energy required for materials to resist catastrophic fracture. Kisailus et al. have found that it can also cause crack twisting, which in turn increases the crack area and enhances energy dissipation [17]. Additionally, other scholars have conducted systematic studies on the angles of the helical structure, exploring optimal models under different angular arrangement patterns [18,19,20,21]. For the impact region, which exhibits the strongest impact resistance, researchers performed cantilever experiments on samples with herringbone fractures and confirmed that plastic dissipation at the crack tip is the core mechanism behind the excellent impact resistance of its sinusoidal structure. Inspired by the sinusoidal structure of the impact region, researchers have designed bionic bidirectional corrugated structures, which effectively improve the energy absorption efficiency of sandwich structures [22]. Combining with the gradient arrangement in the impact region, they have also developed a bionic gradient bidirectional sinusoidal arrangement structure to enhance the impact resistance in the *Z*-axis direction. Furthermore, some studies have coupled the sinusoidal structure with the helical structure to explore their cooperative mechanism: during the impact process, the helical structure can improve the impact toughness of composite materials by dissipating energy, while the sinusoidal structure can disperse and buffer stress, preventing material failure under severe impact due to stress concentration [23,24,25,26]. However, the MSA is a complex and highly efficient three-dimensional structure, and its strong impact resistance stems from the synergistic effect of multiple microstructures. The composition of its gradient components [27], particularly the fiber structure and arrangement in the primary impact-resistant regions, is often the focus of research and application for the superior impact resistance of the MSA. As the region where the MSA plays a dominant role during impact, the interior of the impact zone possesses a complex three-dimensional structure. The comprehensive contribution of these structures to the impact resistance process remains inconclusive to date. Previous research on this region has mostly concentrated on the study and application of its sine-like structure [28], lacking a multi-directional, integrated investigation of both the sinusoidal structure and fiber extrusion in other orientations. In summary, in-depth investigation into the influence of the combination mode of complex three-dimensional microstructures within the impact region on its impact resistance, and the revelation of their synergistic mechanism, hold significant research value; as the core region for impact resistance, systematic study of the functions of various special microstructures in the impact region and their potential for engineering applications also possesses important practical significance.

In this study, the microstructure of the impact region of the MSA is taken as the bionic prototype. Integrating its complex structural characteristics, a fiber structure optimization model with three-dimensional anisotropic design was proposed based on the bionic design concept of integrating strength and toughness. The objective of this research is to reveal the comprehensive relationship between various microstructures of composite materials and their impact performance under dynamic loading conditions, so as to ultimately improve the impact resistance of composite materials. Meanwhile, through analyzing the impact fracture mechanism of the three-dimensional model, theoretical support and a design basis are provided for the further optimization of the impact resistance of bionic composite materials.

## 2. Finite Element Simulation and Low-Velocity Impact Test

### 2.1. Bionic Model Design and FEM

**Figure 1:**
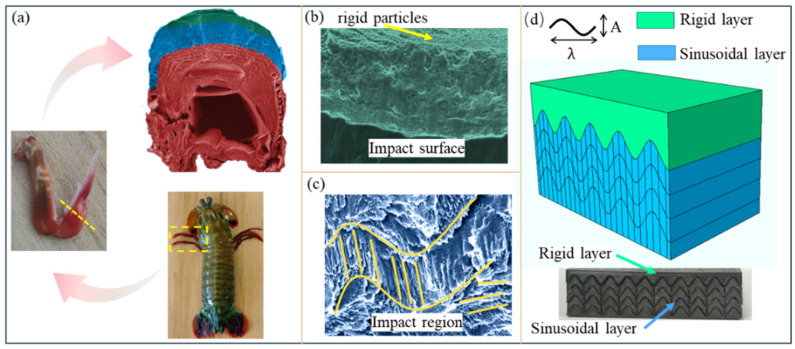
Biology and model structure of the MSA. (**a**) SEM of the MSA. (**b**) High-resolution SEM image of the impact surface. Rigid particles cover its surface. (**c**) High-resolution SEM image of the impact region, including sinusoidal structures, vertical fibers and horizontal fibers. (**d**) Structure composition of bionic model.

This study focuses on the complex microstructure within the impact region of the MSA. The exceptional impact resistance of this impact region mainly stems from two aspects (Figure 1a–c). First, the surface hard nanoparticle layer offers excellent cushioning and protective effects. Second, the sinusoidally arranged mineral fibers in the region, along with interspersed vertical fibers, can effectively suppress violent crack growth [16]. Inspired by this complex microstructure in the impact region, our research team has previously designed a bionic structure consisting of a rigid outer layer and a flexible sinusoidal inner layer based on the structural characteristics of the impact region [29,30], as shown in Figure 1d. The rigid outer layer provides effective protection, while the underlying flexible sinusoidal inner layer can guide the direction of crack propagation and significantly extend the crack path. Additionally, by establishing a cohesive zone model and performing impact simulation tests, we have not only simulated the initiation and propagation processes of cracks in the bionic structure but also verified that the longitudinal fiber arrangement enhances resistance to crack initiation and propagation. However, the previous research had obvious limitations: it was confined to the design of two-dimensional (2D) model structures, making it impossible to ensure that the vertical fibers still maintain strong impact resistance in three-dimensional (3D) models. Nature serves as a rich and efficient source of inspiration for the innovative design of material structures. Thus, integrating multiple biological structures, giving full play to the advantages of each structure, and compensating for the defects of a single structure have become a highly feasible strategy [10,31,32]. To enhance the model’s impact-resistant capability in the 3D direction, this study first performs bionic design on fiber configurations in multiple directions and then realizes performance improvement by exploiting the synergistic effect of these configurations.

**Figure 2:**
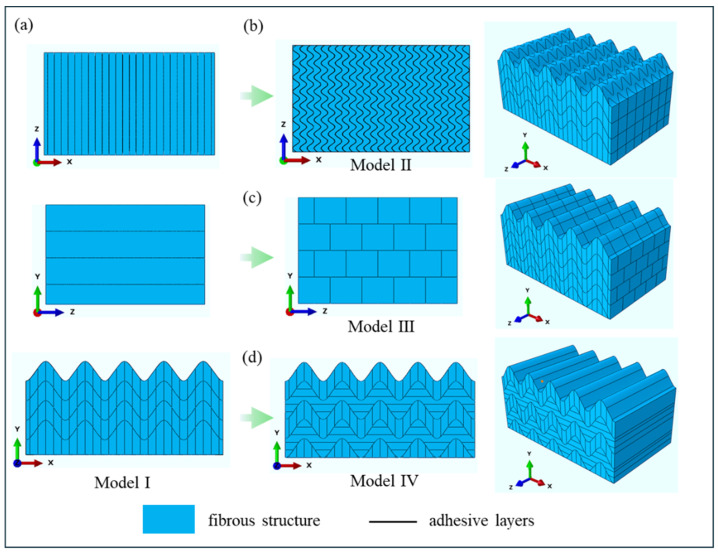
Fiber structure design in three directions for four models. (**a**) the fiber structure of Model I in three directions. (**b**) Sinusoidal vertical fibers in X-Z direction (Model II). (**c**) Brick-and-mortar fibers in Y-Z direction (Model III). (**d**) Vertical and horizontal alternating fibers in X-Y direction (Model IV).

In order to investigate the effect of different fiber arrangements on the 3D directional impact resistance of structures, this study proposed four models with distinct fiber arrangements to verify their impact resistance performance. Specifically, FEM simulations and impact tests were conducted to verify whether the arrangement of multiple fibers in 3D space can still enhance resistance to crack initiation and propagation. All models adopt a composite structure design consisting of an outer rigid layer and an inner sinusoidal layer [33,34], where the sinusoid has an amplitude λ of 0.2 mm and a wavelength A of 0.1 mm. The sinusoidal structure consists of four layers in total. With the exception of the bottom layer, each layer has a thickness of 0.1 mm. An appropriate number of sinusoidal layers enables the full exertion of the stress dispersion capability of the sinusoidal structure under impact loading. Notably, the sinusoidal layers of all models, including Model I, contain vertical fibers aligned along the Y-direction, as shown in Figure 2a. It is noteworthy that, to simulate different fiber orientations, adhesion units with distinct arrangements are configured at the structural junctions to mimic the fibrous structure and fiber directions. The fiber blocks are separated by cohesive elements, resulting in different fiber orientations. For Model II (Figure 2b), to mitigate structural damage and further improve its energy absorption capacity, the planar sheet-like vertical fibers were optimized in 3D. This model was designed by integrating the sinusoidal arrangement structure from the impact region of the MSA; by adjusting the distribution of vertical fibers in the X-Z direction, longitudinally sinusoidal vertical fibers are formed. Transforming linear fibers into sinusoidal fibers in the 3D direction can further leverage the advantages of the sine-wave pattern, including extending the crack propagation path, alleviating stress concentration, and enhancing energy absorption [35]. As shown in Figure 2c, the design concept of Model III involves introducing nacre’s unique brick-and-mortar structure in the Y-Z direction—a structure proven to effectively enhance material strength and toughness [36,37,38]. Derived from Model I, Model IV (Figure 2d) incorporates the alternating fiber distribution trait of the sinusoidal layer in the impact region, and an alternating arrangement of vertical and horizontal fibers is designed in the X-Y direction. This multi-directional alternating fiber layout imparts anisotropic properties and excellent fracture toughness to the material [16].

Implicit analysis algorithm face inherent challenges in simulating material failure during impact loading, as their mathematical framework relies on the continuity assumption of the material continuum during deformation—an assumption that breaks down at the onset of crack initiation and propagation. To bridge this research gap, a variety of fracture-mechanics-based techniques have been proposed, among which the cohesive finite element method (CFEM) has emerged as a highly effective approach for modeling discrete crack growth and interfacial failure in composite materials, including bio-inspired composites [39]. In this paper, the maximum stress criterion is adopted for damage assessment of adhesive materials. The material begins to deform and fail when the stress it bears exceeds the maximum stress *τ_max_*. The damage variable is defined as follows:

(1) $$\delta_{max}=\frac{2G_{c}}{\tau_{max}}$$

where *G_C_* is the critical fracture energy; *δ_max_* is the maximum displacement prior to complete failure. The displacement *δ_f_* corresponding to the maximum stress *τ_max_* is defined as follows:

(2) $$\delta_{f}=\frac{\tau_{max}}{K}$$

To simulate crack initiation, crack propagation, and stress distribution, finite element analysis was conducted on the models, with Abaqus (2023) software employed for dynamic load analysis and calculation. For the 3D models, eight-node linear elements (C3D8) were adopted to ensure calculation accuracy; simultaneously, to simulate the initiation and propagation of cracks under impact loading, zero-thickness cohesive elements (COH3D8) were placed between the C3D8 elements. In addition, a mesh size of 0.01 mm is applied at the impact center and locations with complex structures, while a mesh size of 0.02 mm is adopted elsewhere to ensure computational accuracy and convergence. The adhesion model adopted in this paper is the traction-separation model, where the material separates at the location of maximum stress. The contact condition was set as general contact, and a fixed constraint was applied to the bottom of the models. A rigid spherical ball with an initial velocity of 5 m/s was placed on top of the models, with only the degree of freedom in the Y-direction retained to simulate the low-velocity impact condition. Carbon fiber-reinforced polymer (CFRP) composites have been increasingly adopted in anti-collision components due to their exceptional mechanical properties. PC-CF was used for the rigid outer layer: as an external protective layer, it possesses excellent cushioning performance, which helps reduce the risk of material failure. The flexible inner layer was made of carbon fiber-reinforced PETG-CF composite, which possess outstanding comprehensive mechanical properties. Specific material parameters are listed in Table 1.

The constitutive modeling logic in this study focuses on interface-dominated damage and energy dissipation. The bulk material is treated as a linearly elastic body, and damage nonlinearity is introduced only via interface cohesive elements to simulate the debonding between fibers and matrix as well as crack propagation. Bulk nonlinearities such as plastic flow and rate-dependent deformation within the material are neglected. This assumption is suitable for the preliminary validation of structural design effectiveness. Under low-velocity impact, interface debonding and the guidance of crack propagation via structural geometric regulation are the core factors that distinguish the performance differences among different models. After incorporating bulk nonlinearity, the predicted energy absorption of all models may be closer to experimental values, but the relative performance ranking among different models will most likely remain unchanged. The core advantage of the structural design in this study is the structural enhancement based on geometry and interface mechanisms, which is independent of the material constitutive model type.

### 2.2. Low-Velocity Impact Test

Additive manufacturing (AM), more commonly referred to as 3D printing, has enabled unprecedented design freedom for fabricating components with complex geometries and distinct microstructural features [42]. For further study on the impact resistance of bionic structure laminates, experimental samples were made by FDM printing technology, and the manufacturing error is controlled within 0.2 mm. Drop weight impact tests were conducted to verify the impact resistance of the samples, and the experimental results were cross-validated with simulation results to ensure data reliability. Considering the constraints of material cost and manufacturing process, Polycarbonate Carbon Fiber (PC-CF) is selected for the rigid layer due to its high stiffness, which makes it suitable for serving as the rigid layer. The sinusoidal layer adopts Polyethylene Terephthalate Glycol-modified Carbon Fiber (PETG-CF) with excellent toughness. For different structural units of the simulation model, each structural unit is printed in segments and then bonded using resin adhesive to achieve the structure designed in the simulation. Considering the printing accuracy and practical manufacturing constraints of FDM, the sinusoidal profile was scaled up proportionally, with the wavelength λ of 8 mm and the amplitude A of 4 mm. The printing parameters for the composite filaments were set as follows: for PC-CF, the heated bed temperature was 110 °C and the nozzle extrusion temperature was 270 °C; for PETG-CF, the heated bed temperature was 70 °C, and the nozzle extrusion temperature was 250 °C. The layer height of the rigid structure was 5 mm, while that of the sinusoidal structure was 4 mm.

During the experiments, the specimens were fixed to the impact test bench, with sensors mounted beneath them. The impact hammer acquired an initial velocity via free fall, and its speed was further boosted using a spring; in this accelerated state, the impactor ultimately applied drop weight impact loading to the specimens. Three counterweights were attached to the tail end of the impactor and researchers could adjust the number of counterweights to change the total mass of the impactor, thereby regulating the impact energy. After the impact loading was completed, the sample surfaces were processed, followed by observation and analysis of their macroscopic failure morphology and microscopic damage characteristics. All experiments were performed at room temperature, with key experimental parameters as follows: the impact hammer’s inherent mass was 1 kg; the mass of a single counterweight was 0.2 kg; and the impact speed was 5 m/s, the impact energy is set to 10 J. The drop weight impacts at the center of the X-Y plane of the rigid layer, with the impact direction along the *Z*-axis. Additionally, to prevent specimen displacement during impact, clamping plates secured the specimens to the impact device’s base.

## 3. Results and Discussion

### 3.1. Finite Element Model and Impact Test Results

**Figure 3:**
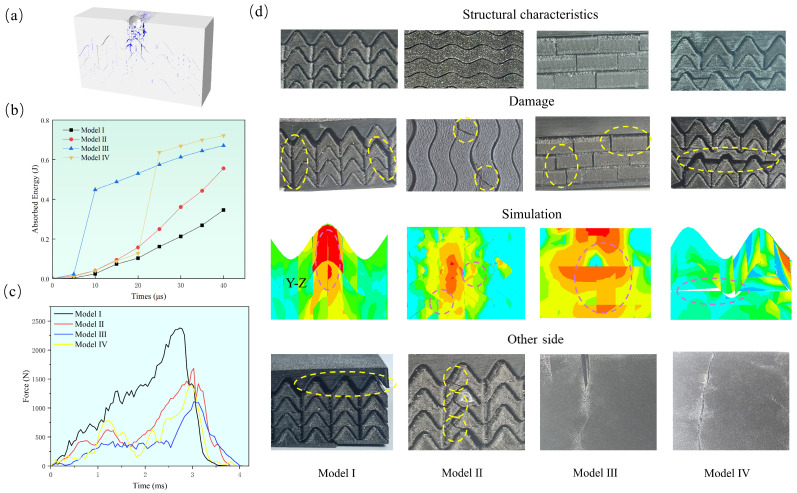
Low-velocity impact experiments and related simulation results. (**a**) Fracture damage of the rigid layer. (**b**) Energy absorption of all models. (**c**) Impact force-time curve of drop weight test. (**d**) Experimental and simulation results on different sides of all models. The area enclosed by the circle is the damaged region.

Figure 3a illustrates the fracture behavior of overall models during the impact process. The rigid layer plays an obvious buffering role: it mitigates the impact force through fragmentation and absorbs a portion of the impact energy. Despite all four models having the same rigid outer layer and similar flexible inner layers, their energy absorption capacities exhibit substantial differences, as shown in Figure 3b. During low-velocity impact, energy dissipation primarily occurs via elastic deformation, plastic deformation, viscoelastic damage, and other damage mechanisms. Accordingly, the total strain energy ALLIE is adopted to characterize the magnitude of energy absorption. Compared with Model I, the other three models exhibit enhanced energy absorption performance, with Model III achieving improvements of 94% and Model IV achieving 109%. In addition, Model III and Model IV each exhibit a period of efficient energy absorption, and these two periods occur at distinct stages during the impact process. The different impact resistance mechanisms of these two models result in distinct impact phenomena.

**Figure 4:**
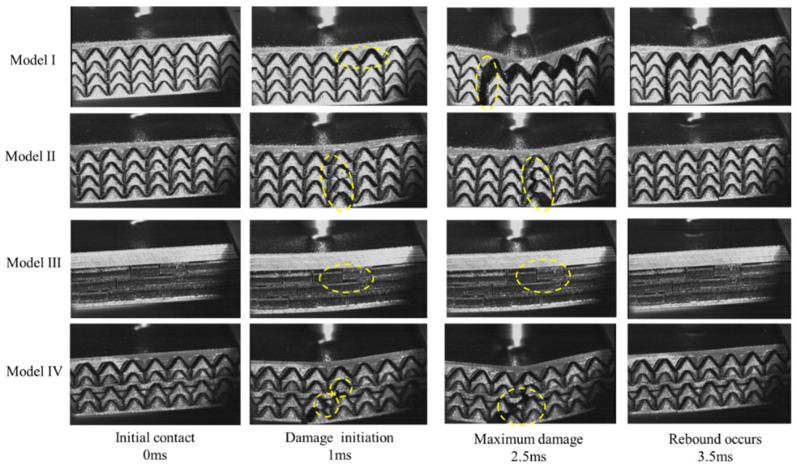
Crack initiation and propagation behavior during impact and the corresponding time instants. The area enclosed by the circle is the damaged region.

Figure 3c,d show the impact force-time curve and morphology of the 3D-printed specimens after low-velocity impact tests, and the experimental results were cross-validated with the simulation outcomes. Model I exhibits the highest peak force followed by a sudden drop, indicating that it has the weakest ability to disperse impact force, and that severe damage and material failure occur distinctly during impact. Model III shows the lowest peak force with minimal damage (Figure 4), compared with Model I, its peak force is reduced by 55%, demonstrating that it possesses the strongest stress dispersion capability and excellent impact resistance. The damage modes of the four models exhibit significant differences. Model I suffers the most severe damage, with catastrophic failure occurring at the impact center, where the surrounding material is almost completely degraded. In contrast, only minor cracks appear on the back face of Models III and IV. For Model II, several microcracks emerge at the ends of the sinusoidal vertical fibers, which alter the propagation path of impact energy and prevent direct material failure. This is also reflected in Figure 4, where the crack propagation path shows a significant directional deflection compared with Model I. According to the simulation results, this phenomenon is attributed to stress relaxation at the ends of the sinusoidal structure. Owing to the dispersion of impact stress, the overall impact fracture performance of Model III is distinctly superior to that of Model I, characterized by smaller and more uniformly distributed cracks. The impact center region of Model I essentially loses its protective function. Conversely, only small cracks are observed at the bottom of Model III, which retains satisfactory protective performance. As for Model IV, although extensive delamination occurs in the horizontal fiber layers, such large-scale delamination inhibits catastrophic failure at the bottom and prevents cracks from propagating directly toward the impact center. It can be concluded from the simulation results that this delamination is induced by the synergistic effect between the horizontal fibers and the sinusoidal structure.

**Figure 5:**
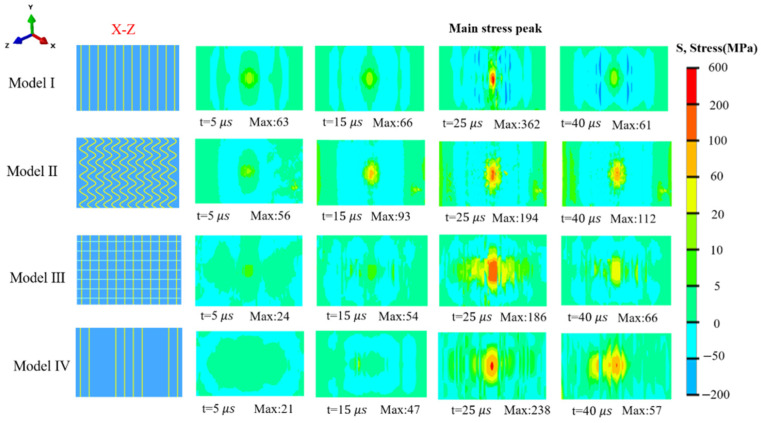
Equivalent nephogram of compressive stress in X-Z plane.

All 3D models exhibit significant disparities in stress distribution characteristics across the three planes (X-Z, Y-Z, X-Y). As shown in Figure 5, on the X-Z plane, Model I exhibits stress concentration in the middle and stress dispersion on both sides, with an obvious fracture failure area. This area appears as a dark blue strip in the stress cloud map, corresponding to a negative compressive stress value. This characteristic indicates that during the impact process of Model I, the material in the middle has reached the failure threshold due to excessive stress concentration, making it prone to fracture damage. When compared to Model I, the stress distribution of Model II on the X-Z plane is more dispersed. It is specifically expressed as stress concentration occurs along the sine tips of the sinusoidal vertical fibers. In numerical terms, the maximum stress in Model II is significantly lower than that in Model I, while the relative area of the high-stress region is larger; this characteristic effectively reduces the risk of overload failure in local materials. Model III is characterized by a relatively uniform stress distribution on the X-Z plane. The phenomenon of high stress concentration is significantly suppressed, and there is no dark blue fracture failure area similar to that in Model I. When Model III is under stress, the stress can be transmitted reasonably through its structural design, greatly reducing the possibility of severe fracture failure of the material. Since the cross-section of Model IV contains both vertical fibers and horizontal fibers, the stress distribution of Model IV on the X-Z plane shows obvious regional differences—the vertical fiber area and the horizontal fiber area exhibit two distinct stress distribution patterns. Compared with Model I, the other models can improve the stress concentration on the X-Z plane and avoid the occurrence of dark blue fracture areas. However, their mechanisms of stress dispersion and impact resistance show distinct differences. Model II leverages the force transmission characteristic of the sinusoidal structure, enabling stress to propagate along the sinusoidal path within the same fiber. Subsequently, stress concentration occurs at the sinusoidal tips, where the stress is simultaneously released. Both Model I and Model II exhibit stress peaks at their centers: Model I shows an elongated shape, while Model II presents sinusoidal block-like patterns. Model III, by virtue of the structural configuration where multiple directions fibers intersect with one another, establishes multi-path channels for stress transmission. When under impact, stress can be transferred and dispersed between different fibers through the intersection nodes, rather than being concentrated in a single fiber or local area. Eventually, a uniform overall stress distribution is achieved. The high-stress area of Model IV is significantly reduced in contrast to Model I. This is attributed to the distinct distribution of the fiber structure in Model IV on the X-Z plane; specifically, the fiber directions differ between adjacent sinusoidal layers. This makes the other side of the vertical fiber at the same height become a horizontal fiber, thereby changing the direction of stress propagation and avoiding fractures caused by simultaneous stress concentration on both sides of the vertical fiber.

**Figure 6:**
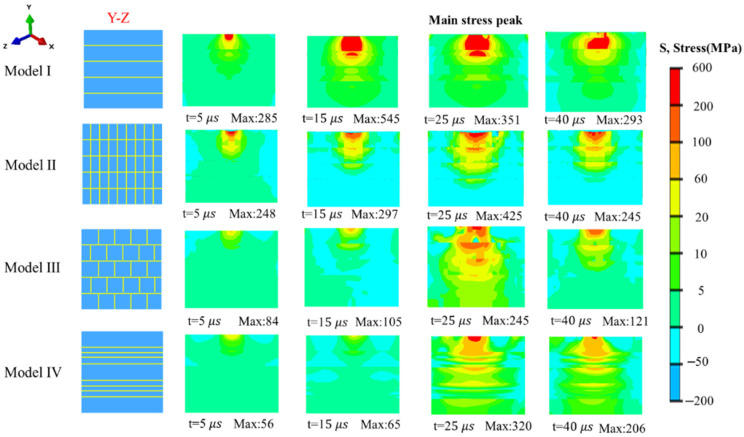
Equivalent nephogram of compressive stress in Y-Z plane.

Figure 6 shows the equivalent nephogram of compressive stress on Y-Z plane. Model I presents a typical simple layered structure on the Y-Z plane, without additional fiber reinforcement. Its most prominent stress characteristic is the occurrence of obvious stress concentration at the interface between the upper and lower layers, along the direction of fiber extension. This phenomenon arises because the layered structure lacks stress transition and dispersion channels. Under loading, the interface acts as a weak link in stress transmission and is susceptible to interface debonding due to local stress overload. Compared with Model I, Model II incorporates sinusoidal vertical fibers, forming a composite structure of simple layers with vertical fibers on the Y-Z plane. This design brings two key improvements to stress distribution: first, the coverage of high stress is significantly reduced, preventing excessive stress concentration; second, the stress exhibits a longitudinal stepwise distribution—the vertical fibers act as longitudinal force-transfer carriers, transmitting stress gradually along the fiber direction, which reduces local stress and avoids sudden stress spikes. Model III features brick-and-mortar structure on the Y-Z plane, where fibers in the upper and lower layers are completely interlaced. This interlaced design provides a critical advantage for stress transmission: when stress propagates from top to bottom, the interlaced fibers force a change in the direction of stress propagation, dispersing stress in multiple directions along the interlaced paths rather than concentrating it in a single channel. The stress concentration regions in Model I exhibit an obvious large-area distribution, whereas those in Model III show a relatively diffuse distribution characterized by a low stress value in the central region and a stepwise downward transmission of stress. This is because stress concentration effects tend to occur at structural discontinuities. For Model I (on the Y-Z plane), there is no direct connection between the upper and lower layers, leading to inevitable stress concentration at the interfaces between layers. In contrast, the brick-and-mortar structure of Model III effectively connects the upper and lower layers, thereby avoiding such a phenomenon. As a result, the stress peak of Model III is significantly lower than other models, making it the optimal solution for stress control on the Y-Z plane. Model IV is distinguished by the addition of numerous horizontal fibers compared to other models. This design is reflected as horizontal differences on the Y-Z plane: multiple layers appear within the horizontal fiber layers. There are obvious differences in stress values between different horizontal fiber layers, forming multiple independent stress-layered regions. The horizontal differences do not significantly improve the impact resistance in this direction.

**Figure 7:**
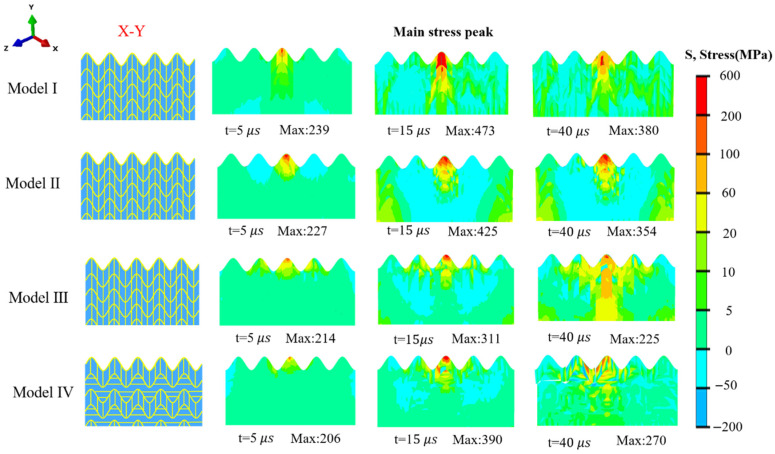
Equivalent nephogram of compressive stress in X-Y plane.

As shown in Figure 7, on the X-Y plane, Model I features a cross-distributed structure consisting of sinusoidal structures and vertical fibers. Its stress transmission exhibits distinct directionality: stress is generated in the impact center area, spreads horizontally along the sinusoidal direction, and is transmitted downward through the vertical fibers. Notably, cracking occurs in the impact center of Model I, and its stress peak is the highest. While Models II and III share the same structure as Model I on the X-Y plane, they differ in 3D fiber design and thus exhibit distinct stress behaviors. Model II uses sinusoidal vertical fibers instead of straight ones. This design allows the fibers to guide stress transmission in both in-plane and out-of-plane directions, preventing stress from concentrating in a single plane. Consequently, Model II shows a reduced peak force and a wider stress propagation range compared to Model I. Model III contains numerous fiber structures that are parallel and perpendicular to the X-Y plane. Therefore, although it has the same cross-section as the other models on the X-Y plane, it possesses more crossed fibers that facilitate stress dispersion—both in the horizontal direction and the in-plane/out-of-plane directions. Model IV is characterized by the alternating distribution of vertical fibers and horizontal fibers between the upper and lower sinusoidal layers. The horizontal fiber region and the vertical fiber region exhibit distinctly different properties: the vertical fibers guide the stress to transmit downward along the fiber direction, while the horizontal fibers undergo severe cracking, followed by large-scale delamination. And a significant reduction in stress is observed after the occurrence of this large-scale delamination. A portion of the sinusoidal structure’s function is to guide stress transmission along the sinusoidal direction, which is generally horizontal—consistent with the force-transmission direction of the horizontal fibers. The accumulation of substantial horizontal stress thus induces cracking and delamination in the horizontal fibers. This large-scale delamination also enables the simultaneous concentrated release of stress and dissipation of significant impact energy. The crack propagation of Model I is that cracks initiate at the crack center and propagate downward along the vertical fibers, while that of Model IV is transverse propagation within the horizontal fiber layers. This is because the stress peak of Model I exceeds the fracture strength of the material, leading to the initiation and propagation of cracks along the shape of the stress peak at the location of the stress concentration. In contrast, after crack initiation in Model IV, the cracks propagate through the horizontal fibers. As a result, after the delamination occurs, the stress concentration inside Model IV is significantly alleviated, thereby protecting the remaining structure from further damage.

**Figure 8:**
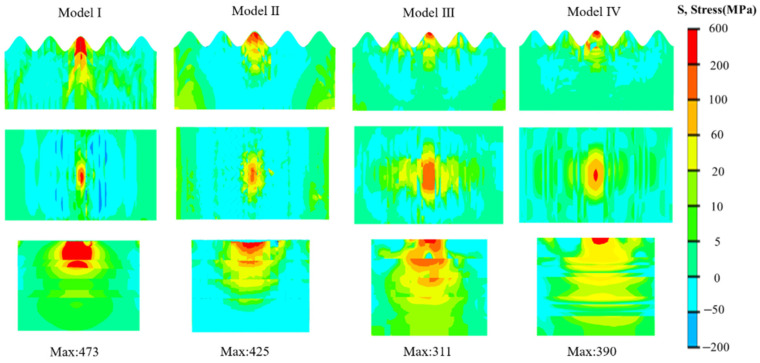
Equivalent nephogram of peak equivalent stress for multi-directional loading.

As shown in Figure 8, Model III exhibits a stress peak far lower than the other models, along with a significantly more uniform stress distribution. Compared with other models, Model III has a larger area of relatively high stress distribution, and the stress values are more uniformly distributed; meanwhile, the distribution area of excessively high stress is smaller than that of other models. The brick-mortar structure combines with vertical fibers to form a stress transfer network in different directions. This uniform stress state effectively reduces the risk of local overload and endows Model III with higher fracture toughness.

### 3.2. Analysis of Impact Resistance Mechanism

**Figure 9:**
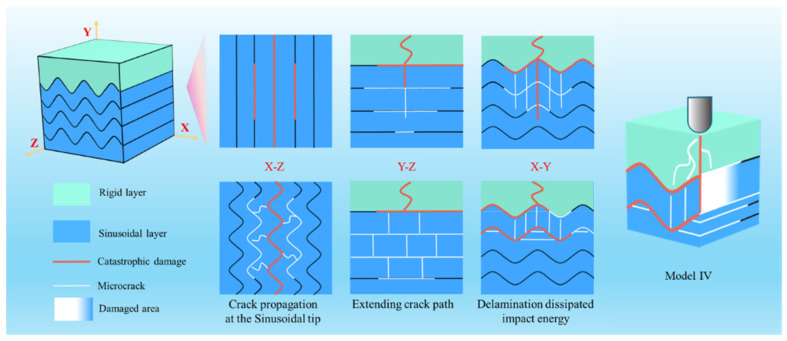
Paths and principle of stress transfer and crack propagation.

Based on the Regularities revealed by finite element simulations and impact tests, three integrated bionic structures are proposed to enhance the comprehensive mechanical properties of composite materials. The quantitative comparison results show that, taking Model I as the benchmark, the maximum stress of Model II is reduced by 10% and the energy absorption is increased by 55%; the maximum stress of Model III is reduced by up to 35% with a 94% improvement in energy absorption; for Model IV, the maximum stress is decreased by 18%, and its energy absorption is enhanced by the highest margin, reaching 109%. Considering both the stress level and energy absorption characteristics comprehensively, Model III exhibits the optimal impact resistance and is the preferred solution for the design of impact-resistant composite structures. As shown in Figure 9, the impact resistance of these three bionic structures can be explained from the following aspects. First, the rigid outer layer generates cracks and fragments through fragmentation; these dissipate part of the impact energy and serve as the first line of defense against impact loads. Subsequently, the flexible inner layer guides the direction of stress transmission via the sinusoidal structure and vertical fibers, thereby mitigating the risk of catastrophic material failure. Second, compared with ordinary vertical fibers, sinusoidal vertical fibers exhibit superior stress dispersion capability while large-scale damage will still occur at the impact center. The combination of the brick-and-mortar structure with vertical fibers and sinusoidal structures significantly increases the cross-arrangement of fibers, thereby expanding stress distribution paths, increasing the crack area, and reducing the extent of damage caused by cracks. This characteristic makes it more suitable for structures that require avoiding excessive damage during impact. In contrast, the integration of horizontal fibers with the sinusoidal structure substantially increases horizontal stress, leading to large-scale delamination in the horizontal direction and thus dissipating a considerable amount of energy. In general, the unique structures of these three models, in synergy with the fiber structure of Model I, collectively contribute to improving the impact resistance of composite materials.

According to the stress distributions along various directions and the features of crack initiation mentioned above, the efficient energy absorption of Model III stems from its brick-and-mortar structure, which enables extensive stress dispersion. While reducing the peak force, this structural synergy generates numerous microcracks; the expanded total area of these cracks ultimately enhances energy absorption. For Model IV, the presence of horizontal fibers leads to large-scale delamination—both horizontally within the horizontal fiber layers and between adjacent sinusoidal layers. This delamination-induced fracture significantly dissipates impact energy. In contrast, the energy absorption of Model I is dominated by only two mechanisms: the fragmentation of the rigid layer and the elastic deformation of the sinusoidal matrix. The energy absorbed by the fragmentation of the rigid layer accounts for approximately 40% of the total. The vertical fibers only bear axial stress and cannot effectively dissipate shear energy, resulting in limited overall energy absorption efficiency. For Model II, the sinusoidal vertical fibers guide stress to propagate along a wave-like path and disperse it to the fiber tips, where energy is absorbed through micro-plastic deformation at the tips, thereby avoiding localized concentrated fracture. However, the load transfer between fibers is relatively weak, leading to a limited improvement in energy absorption.

The sinusoidal layer of Model I consists of a sine-shaped matrix and unidirectional vertical fibers. Although the sinusoidal matrix can disperse the load to a certain extent through its wavy structure, the vertical fibers can only bear axial stress and cannot effectively transfer or offset transverse and shear stresses. This leads to stress easily concentrating at the interface between the vertical fibers and the matrix or in the matrix area of fiber gaps. Such stress concentration will quickly induce catastrophic cracks, ultimately resulting in the overall failure of the structure. Compared with Model I, in Model II, the vertical fibers are designed into a sine-wave shape within the X-Z plane, enabling the sinusoidal structural form and the fiber reinforcement phase to achieve integrated fusion. This integrated structure can form a synergistic effect through the geometric characteristics of the sinusoidal waveform and the reinforcing effect of the fibers, which significantly enhances the vertical fibers’ ability to release locally concentrated stress, effectively inhibits the initiation and propagation of catastrophic cracks in the impact center area under impact load, and reduces the risk of structural failure. However, due to the relative independence of its vertical fibers and the limited number of vertical fibers involved in impact resistance, Model II still exhibits an obvious stress concentration phenomenon in the impact center, and the overall energy dissipation of the model is also lower than Model III and Model IV. Building on Model 1, Model III introduces a brick-and-mortar structure as the third type of reinforcing phase, forming a multi-directional composite reinforcement system. From a spatial perspective, there are significant differences in the stress-bearing directions of the three types of structures: the sinusoidal structure mainly bears loads in the X-Y plane, the vertical fibers focus on axial stress transfer in the Y-Z plane, and the brick-and-mortar structure can simultaneously meet the stress-bearing requirements of both the X-Y and Y-Z planes. This multi-directional reinforcement design constructs a global stress transfer network, which greatly enhances the overall stress dispersion capacity of the structure, significantly reduces the peak stress level inside the structure, and further optimizes the crack resistance. Model IV exhibits stronger impact resistance and protective capabilities compared to other models. This is due to the addition of horizontal fibers, which cause impact cracks to deflect direction. Cracks propagate downward through the rigid layer and reach the sinusoidal layer; within the sinusoidal layer, three types of fibers—sinusoidal fibers, vertical fibers, and horizontal fibers—are arranged alternately, changing the crack direction layer by layer to achieve the effect of rapidly dissipating large amounts of impact energy. While achieving energy dissipation, it effectively blocks the transfer of damage to the underlying material, thereby protecting the underlying structure from severe damage.

In summary, the unidirectional fiber arrangements exhibit inferior impact resistance, while the hybrid fiber layouts realize a substantial enhancement in performance via structural synergy. Among these configurations, Model III presents a more homogeneous stress distribution and preserves structural integrity through a controlled failure mechanism that induces extensive crack propagation; this renders it particularly suitable for scenarios demanding stringent damage control. In contrast, Model IV yields the highest energy absorption efficiency: it modulates the crack propagation direction by virtue of horizontal fibers, thereby maintaining the relative structural integrity of the basal region.

It should be acknowledged that the bionic design of these structures fails to accurately replicate the structure of the impact region, as the impact region of the MSA is far more complex and exhibits higher efficiency. There are also certain deviations between simulation and experiment, but the comparison between the two can still illustrate the differences among different impact-resistant structures. Consequently, future work is expected to focus on establishing more precise and efficient bionic models, which can be applied to the design and manufacturing of practical impact-resistant materials. In terms of scalability, the structural design is independent of specific dimensions or materials; by adjusting the parameters of the sinusoidal structure and fiber arrangement density, it can adapt to the dimensional requirements of components ranging from micro precision parts to large-scale industrial components. Meanwhile, the materials of the rigid and flexible layers can be replaced with aerospace-grade carbon fiber, metal matrix composites and other advanced materials to meet the performance requirements of different application scenarios. In terms of industrial applicability, the FDM 3D printing combined with the resin bonding process adopted in the model is highly compatible with existing industrial production lines. The mass production of complex fiber arrangements can be realized via automated fiber placement technology, and the raw materials are commonly used in industry with controllable costs. Although challenges such as fiber arrangement accuracy and interlaminar bonding strength may be encountered in large-scale production, these issues can be addressed by optimizing manufacturing processes and material formulations. In summary, the proposed bionic structure not only demonstrates excellent impact resistance at the laboratory scale but also possesses great potential for translation to industrial applications.

## 4. Conclusions

Based on the microstructures of the mantis shrimp appendage, this study proposes four rigid-flexible coupled bionic structures composed of various fiber arrangements. The results show that the top rigid layer exerts an outstanding buffering effect, thereby preventing the entire model from direct penetration. Due to differences in fiber arrangement, the stress distribution and energy absorption performance of each model vary. The sinusoidal arrangement of fibers enables stress dispersion, guides the transfer of stress and cracks along the sinusoidal path, and achieves energy dissipation at the crack tip, thereby significantly extending the crack propagation path. Furthermore, composite structures with multiple special fiber arrangements exhibit remarkably superior impact resistance compared to structures with a single type of fiber arrangement. Specifically, the combination of the sinusoidal structure and the brick-and-mortar structure can greatly enhance stress dispersion efficiency, enabling it to effectively reduce the peak force and effectively prevent structural failure caused by severe damage. Meanwhile, the alternating layout of vertical and horizontal fibers can alter the direction of crack propagation, improve the energy absorption capacity, and thus strengthen the structure’s protective performance. As a result, the bottom cracks of both structures are relatively small after impact, and they still retain a relatively intact protective capability. Given that the composite arrangement of multiple fiber structures can significantly enhance impact resistance under low-velocity impact, this study offers new insights and theoretical references for the structural design of impact-resistant materials applied to special and critical components in the aerospace, automotive, and marine sectors.

## Figures and Tables

**Table 1:** Parameters of bionic model [29,40,41]: ρ denotes density; E_11_, E_22_, and E_33_ are the elastic moduli in three directions; υ is the Poisson’s ratio; K is the ratio of the maximum stress τ_max_ to the corresponding displacement δ_f_; and GC represents the fracture energy.

Materials	ρ (kg/m^3^)	E_11_ (GPa)	E_22_	E_33_	υ	K (MPa/mm)	τ_max_ (MPa)	G_C_ (J/m^2^)
PC-CF	1180	25.0	3.8	3.8	0.34		200	6500
PC-CF interface	1180				0.34	1.8 × 10^6^	22.5	520
PETG-CF	1170	8.5	3.2	3.2	0.35		280	9200
PETG-CF interface	1170				0.35	1.5 × 10^6^	15.8	410

## Data Availability

The datasets generated and analyzed during the current study are available from the corresponding authors upon reasonable request.
